# The gut–kidney axis is regulated by astragaloside IV to inhibit cyclosporine A-induced nephrotoxicity

**DOI:** 10.3389/fphar.2025.1518481

**Published:** 2025-01-27

**Authors:** Cong Han, Ran-ran Gao, Le Zhou, Wei Li

**Affiliations:** ^1^ Nephropathy Department, Affiliated Hospital of Shandong University of Traditional Chinese Medicine, Jinan, China; ^2^ College of First Clinical Medicine, Shandong University of Traditional Chinese Medicine, Jinan, China

**Keywords:** astragaloside IV, cyclosporine-induced nephrotoxicity, sequencing of 16S rDNA, LC-MS analysis, sequencing of lncRNAs and mRNAs

## Abstract

**Introduction:**

Chronic nephrotoxicity caused by CNIs (CICN) manifests clinically as chronic kidney disease (CKD). Astragaloside IV (AS-IV) plays a certain role in the treatment of CKD. This study aimed to verify the ameliorative effects of AS-IV on CICN and further explore the mechanisms underlying the modulation of the “gut–transcriptome–metabolome coexpression network” by AS-IV within the context of the “gut–kidney axis” to improve CICN.

**Methods:**

Five groups of 40 mice were studied: a normal group (N, olive oil), a model group (M, CsA, 30 mg kg^-−1^ d^−1^), a low-dose AS-IV group (CsA + AS-IV, 30 mg kg^−1^ d^−1^ + 10 mg kg^−1^ d^−1^), a high-dose AS-IV group (CsA + AS-IV, 30 mg kg^−1^ d^−1^ + 20 mg kg^−1^ d^−1^), and a valsartan group (CsA + Val, 30 mg kg^−1^ d^−1^ + 10 mg kg^−1^ d^−1^). The gut microbiota, renal transcriptome, and urine metabolome were separately detected to construct a gut–transcriptome–metabolome coexpression network. The target species, target genes, and target metabolites of AS-IV were evaluated.

**Results:**

CsA led to increased proteinuria and a deterioration of kidney function, accompanied by increased inflammation and oxidative stress, whereas AS-IV improved kidney damage. AS-IV inhibited intestinal permeability and disrupted the microbiota structure, increasing the abundance of *Lactobacillus reuteri*, *Bifidobacterium animalis*, *Ignatzschineria indica*, and *Blautia glucerasea.* Six coexpression pathways related to transcription and metabolism, including the *citrate cycle*, *ascorbate and aldarate metabolism*, *proximal tubule bicarbonate reclamation*, *glycolysis/gluconeogenesis, ferroptosis,* and *drug metabolism–cytochrome P450*, were identified. Seven target metabolites of AS-IV were identified in the 6 pathways, including UDP-D-galacturonic acid, 2-phenylethanol glucuronide, dehydroascorbic acid, isopentenyl pyrophosphate, alpha-D-glucose, 3-carboxy-1-hydroxypropylthiamine diphosphate and citalopram aldehyde. Five target genes of AS-IV, Ugt1a2, Ugt1a9, Ugt1a5, Pck1, and Slc7a11, were also identified and predicted by NONMMUT144584.1, MSTRG.30357.1 and ENSMUST00000174821. *Lactobacillus reuteri* was highly correlated with renal function and the target genes and metabolites of AS-IV. The target genes and metabolites of AS-IV were further validated. AS-IV inhibited intestinal-derived urinary toxins and improved renal tissue apoptosis, lipid accumulation, collagen deposition, and mitochondrial damage.

**Conclusion:**

AS-IV improved CICN through the coexpression of the gut–transcriptome–metabolome network. The six pathways related to energy metabolism driven by *L. reuteri*, including the *citrate cycle*, *ascorbate and alderate metabolism*, *proximal tube bicarbonate metabolism*, *glycolysis/gluconeogenesis, ferroptosis, drug metabolism–cytochrome P450*, are important mechanisms.

## 1 Introduction

Cyclosporine A (CsA) is a calcium-dependent phosphatase inhibitor that has been shown to be effective in treating several chronic kidney diseases (CKDs). However, its chronic nephrotoxicity has become the most common and serious problem limiting its clinical application and is also an important cause of end-stage renal disease (ESRD) (Yu et al., 2023). The clinical manifestation of chronic nephrotoxicity induced by CNIs (CICN) is CKD, with renal fibrosis (RF) being the main pathological change (El Hennawy et al., 2021). Although certain natural medicines or angiotensin receptor blockers (ARBs) can moderately control CICN, they still cannot prevent its progression (Ghafil et al., 2022; Ibrahim et al., 2022). Therefore, raising awareness of CICN and actively seeking effective prevention and treatment strategies have important clinical and social significance.

The pathogenesis of CICN is complex and involves various aspects, such as the endoplasmic reticulum, oxidative stress, inflammation, autophagy, apoptosis, and lipid metabolism (Wu and Kuca, 2019; Wu et al., 2018). Recent research has revealed a close relationship between the progression of CKD and the gut‒kidney axis. Dysregulation of the intestinal microbiota can lead to the accumulation of uraemic toxins and systemic microinflammatory responses, which impair renal function (Wang et al., 2020; Glorieux et al., 2020). Metabolic waste from the kidneys can further exacerbate intestinal damage, creating a vicious cycle between the two organs (Huang and Chen, 2024; Tong et al., 2024). Long noncoding RNAs (lncRNAs), a type of RNA that does not encode proteins, may regulate the expression of target genes in various diseases, including CKD (van Zonneveld et al., 2023; Ramanathan et al., 2024). Moreover, changes in the gut microbiota can influence the expression of lncRNAs and miRNAs in mice (Zhou et al., 2020). Urine metabolomics is an important tool for reflecting renal metabolic function, with metabolites such as NAG and ACR in urine being able to indicate renal damage in different parts of the body (Wang et al., 2019; Dubin and Rhee, 2020). By analysing changes in urinary metabolites, we can gain a deeper understanding of the pathological mechanisms of CKD. In summary, we hypothesize that gut–transcriptome–metabolome coexpression plays a significant role in CICN, although this hypothesis has not yet been confirmed by research.

In traditional Chinese medicine, CICN is believed to be caused by a deficiency of spleen and kidney qi. *Astragalus membranaceus* (AM), a classic herbal medicine for tonifying qi, exerts renoprotective effects in the clinic (Chan et al., 2024). Astragaloside IV (AS-IV) is the primary active constituent of AM. Studies have shown that AS-IV can ameliorate CKD by modulating the gut microbiota, inhibiting inflammation and oxidative stress, and reducing ferroptosis and endoplasmic reticulum stress (Gao et al., 2023; Shi et al., 2024). Our previous research indicated that AM can ameliorate renal damage induced by hyperglycaemia through the “gut‒kidney axis” (Shen et al., 2023). However, the mechanism by which AS-IV improves CICN by regulating the “gut‒kidney axis” has not been explored.

The purpose of this study was to examine the mechanism by which AS-IV improves CICN by regulating the coexpression of genes involved in the gut–transcriptome–metabolome network utilizing 16rDNA sequencing, urine UHPLC-MS/MS, and renal lncRNA‒mRNA cosequencing technologies.

## 2 Materials and methods

### 2.1 Chemicals and reagents

AS-IV **(**molecular weight 784.97, molecule C_41_H_68_O_14_, purity >97.1%) was purchased from Yuanye Biotechnology Co. Ltd. (Shanghai, China) and was dissolved in 0.5% carboxymethyl cellulose sodium buffer (CMC). Cyclosporine capsules (Batch No. H10960123, Hangzhou Sinopharm East China Pharmaceutical Co., Ltd., Hangzhou, China) were used. A PAS dye solution set(G1008, Servicebio, Wuhan, China) was used. A haematoxylin‒eosin (H&E) HD Constant dye kit (G1076, Servicebio, Wuhan, China) was used. The Masson’s trichrome dye solution was purchased from G1006 (Servicebio, Wuhan, China). Oil Red O solution (G1015, Servicebio, Wuhan, China) was used. Alcian blue dye (G1027, Servicebio, Wuhan, China) was used. A TUNEL assay kit and DAPI (G1502, Servicebio, Wuhan, China) were used. An interleukin-6 (IL-6) kit (230321KE3, Jingmei Biological Technology Co., Ltd., Jiangsu, China) was used. An anti-ACTIN antibody (GB15003, Servicebio, Wuhan, China), anti-UGT1A9 antibody (ab88517, abcam, Cambridge, MA), anti-UGT1A5 antibody (TA360449, OriGene, Wuxi, China), anti-SLC7A11 antibody (GB115276, Servicebio, Wuhan, China), and anti-PCK1 antibody (GB11966, Servicebio, Wuhan, China) were used.

### 2.2 Drug administration and design of animal experiments

A total of 40 C57BL/6 male mice were purchased from Beijing Weishang Lituo Technology Co., Ltd. (age 6–9 weeks, licence SCXK 2021-0010, grade SPF). The Affiliated Hospital of Shandong University of Traditional Chinese Medicine Animal Experiment Ethics Review Committee approved the experiment. The 40 mice were divided into five groups: a normal group (N, olive oil), model group (M, CsA, diluted in olive oil, 30 mg kg^−1^ d^−1^), low-dose AS-IV group (CsA + AS-IV, 30 mg kg^−1^ d^−1^ + 10 mg kg^−1^ d^−1^), high-dose AS-IV group (CsA + AS-IV, 30 mg kg^−1^ d^−1^ + 20 mg kg^−1^ d^−1^), and valsartan group (CsA + Val, 30 mg kg^−1^ d^−1^ + 10 mg kg^−1^ d^−1^). The mice were administered the compounds by gavage for 6 weeks, after which blood, urine, faeces, and colon and kidney tissues were collected.

### 2.3 Biochemical analysis of samples

Serum Scr, BUN, UA, FFA, and UACR levels were detected through biochemical analyses. Serum MDA, SOD, IL-6, PCS, IS and urinary NAG levels were detected by the ELISAs. The kits contained instructions for testing the serum and urine samples.

### 2.4 PAS, HE, Masson’s trichrome, Oil Red O and alcian blue staining

Paraformaldehyde-fixed kidney and colon tissues were embedded in paraffin. We stained paraffin sections with periodic acid–Schiff–haematoxylin, Masson’s trichrome staining solution, haematoxylin–eosin or Oil Red O dye solution–haematoxylin were used to stain the renal tissue. An optical microscope was used to examine paraffin sections of colon tissue stained with haematoxylin–eosin or alcian blue.

### 2.5 Immunofluorescence staining

After antigen retrieval, fluorescence quenching, and blocking with serum, renal tissue sections were incubated with primary antibodies against FN, COL1A1, COL3A1 and TGFβ and the corresponding secondary antibodies. DAPI was used as a counterstain for the nuclei. Fluorescence microscopy was used to examine the slices after sealing.

### 2.6 TUNEL staining of kidney tissue

The paraffin sections were repaired, and a break was made in the membrane. For the tissue sections, reagent 1 (dUTP) and reagent 2 (TdT) were combined in proportion. After adding DAPI staining solution and sealing the cells with an antifluorescence quencher, fluorescence microscopy was used to observe the cells. Yellow nuclei were positive for apoptosis.

### 2.7 Transmission electron microscopy

Kidney tissue was collected, added to electron microscopy fixative, pre-embedded in agar and fixed. After dehydration at room temperature and infiltration embedding, the samples were polymerized, sliced, stained, and observed under a transmission electron microscope.

### 2.8 Sequencing of 16S rDNA

DNA was extracted from faeces, and the 16S V3 + V4 region was amplified by PCR. The PCR product was purified, and a library was constructed and sequenced. The raw data obtained from sequencing were spliced and filtered to obtain valid data. Then, based on the valid data, Uparsene software was used to cluster the sequences into OTUs with a default consistency of 97%, and species annotation was performed. The Shannon index was analysed using R software, and PCoA was performed based on the Euclidean distance. The top 10 genera and species were screened, and the biomarkers with significant differences between groups were compared via LEfSe. The correlations between the gut microbiota and renal function indicators, metabolites, and genes were calculated through Pearson’s correlation analysis. The sequencing procedure was commissioned by Beijing Novogene Technology Co., Ltd.

### 2.9 Urine analysis by UHPLC‒MS/MS

Metabolites were detected using an ultrahigh-performance liquid chromatography system coupled with a high-resolution mass spectrometer. ACQUITY UPLC HSS T3 (100 mm × 2.1 mm, 1.8 um) was used as the chromatographic column. The mobile phases used were B–acetonitrile and A–water (containing 0.1% formic acid). Separation was implemented at a flow rate of 0.35 mL/min and a volume of 3 μL. The mass spectrometer was operated in positive mode with an ion jet voltage of 3800 V, whereas it was operated in negative mode with a −3000 V ion jet voltage. Mass scanning was performed at a frequency of 100–1,200 Hz with ion source temperatures of 550°C (+) and 550°C (−).

Progenesis QI v3.0 software was used to filter baselines, identify peaks, integrate data, correct retention times, align peaks, and normalize the data obtained from mass spectrometry detection. Using the Human Metabolome Database (HMDB), Lipidmaps (v2.3), METLIN database, and EMDB2.0, compounds were identified by their mass numbers, secondary fragments, and isotope distributions. According to OPLS-DA, overall differences in metabolic profiles were observed between the groups. We used OPLS-DA models with VIP values greater than 1 and p values lower than 0.05 for the t-test to identify differentially abundant metabolites. The Pearson correlation coefficient was calculated to evaluate the correlations between differentially abundant metabolites, and a ROC curve was constructed to evaluate the predictability of biomarkers. Analyses of enriched metabolic pathways based on the KEGG database were conducted on differentially abundant metabolites. Shanghai Lu-Ming Biotech Co., Ltd. (China) conducted the entire detection process.

### 2.10 Sequencing and analysis of the renal transcriptome

Total RNA was extracted and quantified. The library was constructed, and its size was detected using an Agilent 2,100 system. Cluster generation was completed on a cBot equipped with an Illumina NovaSeq 6000 sequencer, and subsequent double-ended (PE) sequencing was subsequently performed.

The sequencing results were evaluated for quality using FastQC software. The sequences were filtered with Fastp software to obtain clean reads, and the sequences were compared using HISAT2 software. The FPKM values of genes were calculated using Stringtie software and the TMM algorithm and then quantified for lncRNAs and mRNAs using edgeR. Differentially expressed genes were screened by a P value <0.05 and FC > 2 and are displayed in the form of scatter plots or volcano plots. The target mRNAs of the lncRNAs were predicted in cis and trans, and a coexpression network was constructed. A KEGG enrichment analysis was conducted to identify differentially expressed mRNAs. We worked with Shanghai Whale Boat Gene Technology Co., Ltd., to complete the sequencing.

### 2.11 lncRNA and mRNA analyses using RT‒qPCR

A total RNA extract was obtained and reverse transcribed, after which RT‒qPCR was performed. An instrument from Roche (Germany) was used for all reactions. We standardized the mRNA expression to GAPDH expression levels and performed relative quantification using the 2^−ΔΔCT^ method. The gene-specific primer sequences for 3 lncRNAs and 5 mRNAs (Seville, China) can be found in Supplementary Document 1.

### 2.12 Western blot analysis

A 1% PMSF solution was added to the RIPA lysis buffer before the kidneys were homogenized. The protein concentration was subsequently determined using an enhanced BCA protein assay kit. SDS‒PAGE was subsequently used to separate the proteins, which were subsequently transferred to a PVDF membrane. A 30-min incubation in Tris buffer containing 0.1% Tween-20 was followed by an incubation with the primary antibodies (Pck1, Ugt1a5, Ugt1a9, and Slc7a11) at 4°C. Using an ECL protein detection kit, the protein signal was detected after 30 min of incubation with HRP-labelled goat anti-rabbit IgG. Using ImageJ software, the beta-actin signal, which served as the standard reference signal, and target protein signals were quantified.

### 2.13 Analyses of data

The data were analysed using SPSS 26.0 statistical software (Chicago, United States). The intergroup differences were analysed using one-way ANOVA, with *P* < 0.05 indicating significant differences.

## 3 Results and discussion

### 3.1 AS-IV protected kidney function

**FIGURE 1:**
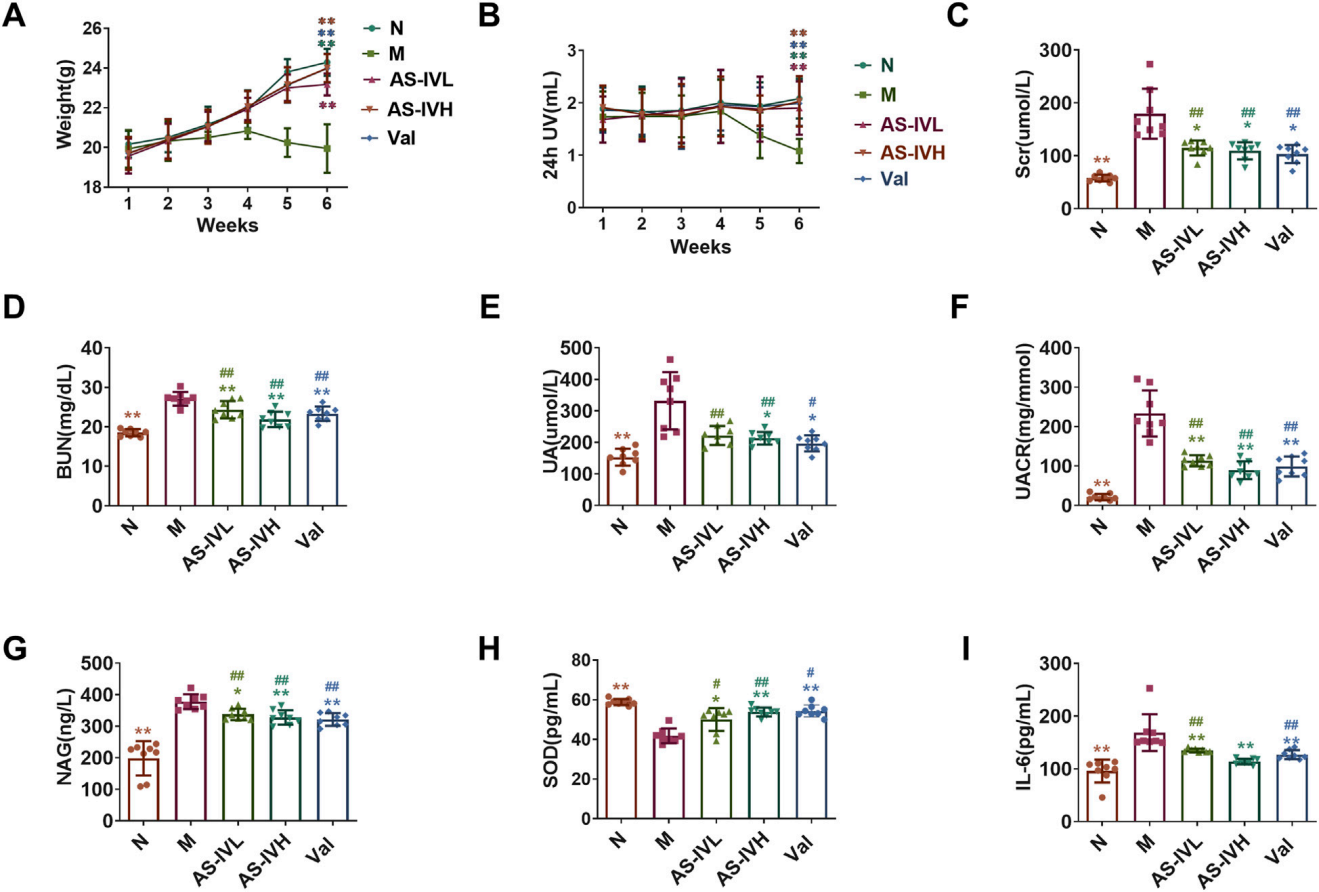
Regulation of renal function by AS-IV. **(A, B)** Weight and 24-h urine volume (UV); **(C–I)** serum creatinine (Scr) levels, blood urea nitrogen (BUN) levels, uric acid (UA) levels, urinary albumin–creatinine ratio (UACR), N-acetyl-β-glucosaminidase (NAG) levels, superoxide dismutase (SOD) levels and interleukin 6 (IL-6) levels are shown. *P < 0.05 and **P < 0.01 compared with the M group. ^#^ *P* < 0.05 and ^##^ *P* < 0.01 compared with Group N. The data are presented as the means ± SDs. (n = 8).

Compared with those in Group N, the body weights and urine outputs of the mice in Group M were lower, whereas AS-IV increased the urine output. There was a significant difference in the sixth week (*P* < 0.01) (Figures 1A, B). The levels of Scr, BUN, UA, UACR, NAG, and IL-6 were significantly higher in Group M than in Group N, whereas the level of SOD was significantly lower (*P* < 0.01). Compared with those in Group M, AS-IV reduced the Scr, BUN, UA, UACR, NAG, and IL-6 levels, and increased SOD levels (*P* < 0.01 or *P* < 0.05) (Figures 1C–I). AS-IV protected kidney function, ameliorated oxidative stress and the microinflammatory status in the body, and the treatment effect of AS-IVH was better.

### 3.2 AS-IV improved kidney and colon pathology

**FIGURE 2:**
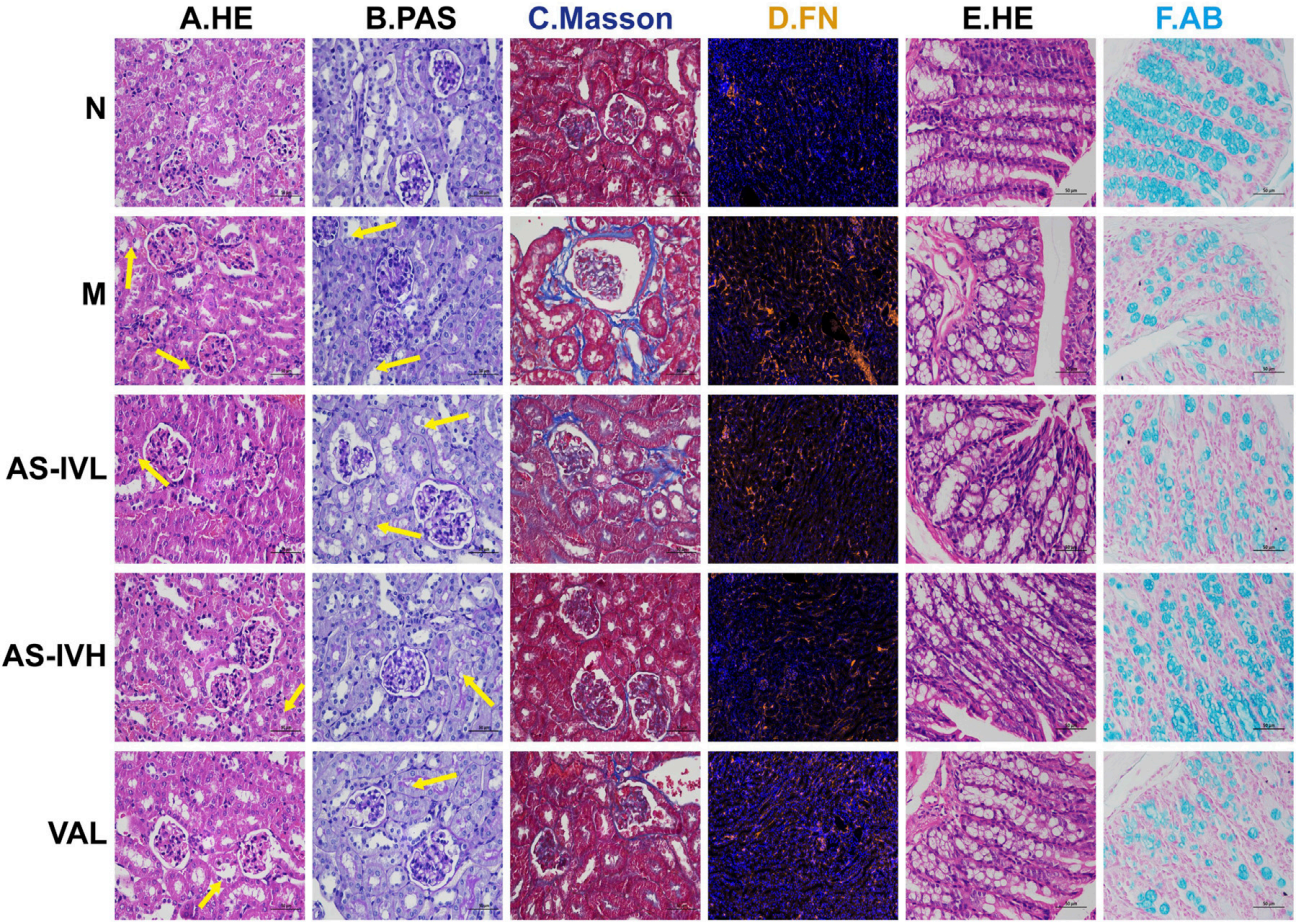
The impact of AS-IV on renal and colon pathology. **(A, B)** HE and PAS staining of renal tissue; the arrow represents the lesion. **(C)** Masson’s trichrome staining of renal tissue. **(D)** Immunofluorescence staining for fibronectin (FN) (brown represents FN). **(E)** HE staining of the colon. **(F)** Alcian blue staining of the colon. The magnification of all the images is ×400.

HE staining revealed significant epithelial cell oedema in the renal tubules of the M group, with swollen cells and a loosely stained cytoplasm that appeared pale. PAS staining showed incomplete or absent brush borders in the renal tubules of the M group. Masson’s trichrome staining demonstrated fibrous tissue proliferation. Following the AS-IV intervention, these pathological injuries were alleviated to varying degrees (Figures 2A–C). Compared with that in Group N, the expression of FN was higher in Group M but decreased following the AS-IV intervention (Figure 2D). Although no obvious inflammation was detected in the intestinal tract of the M group, the intestinal permeability of the M group increased, and AS-IV inhibited intestinal leakage (Figures 2E, F). In the AS-IV group, the pathological damage to the kidney and colon was effectively reduced.

### 3.3 AS-IV regulated the gut microbiota

**FIGURE 3:**
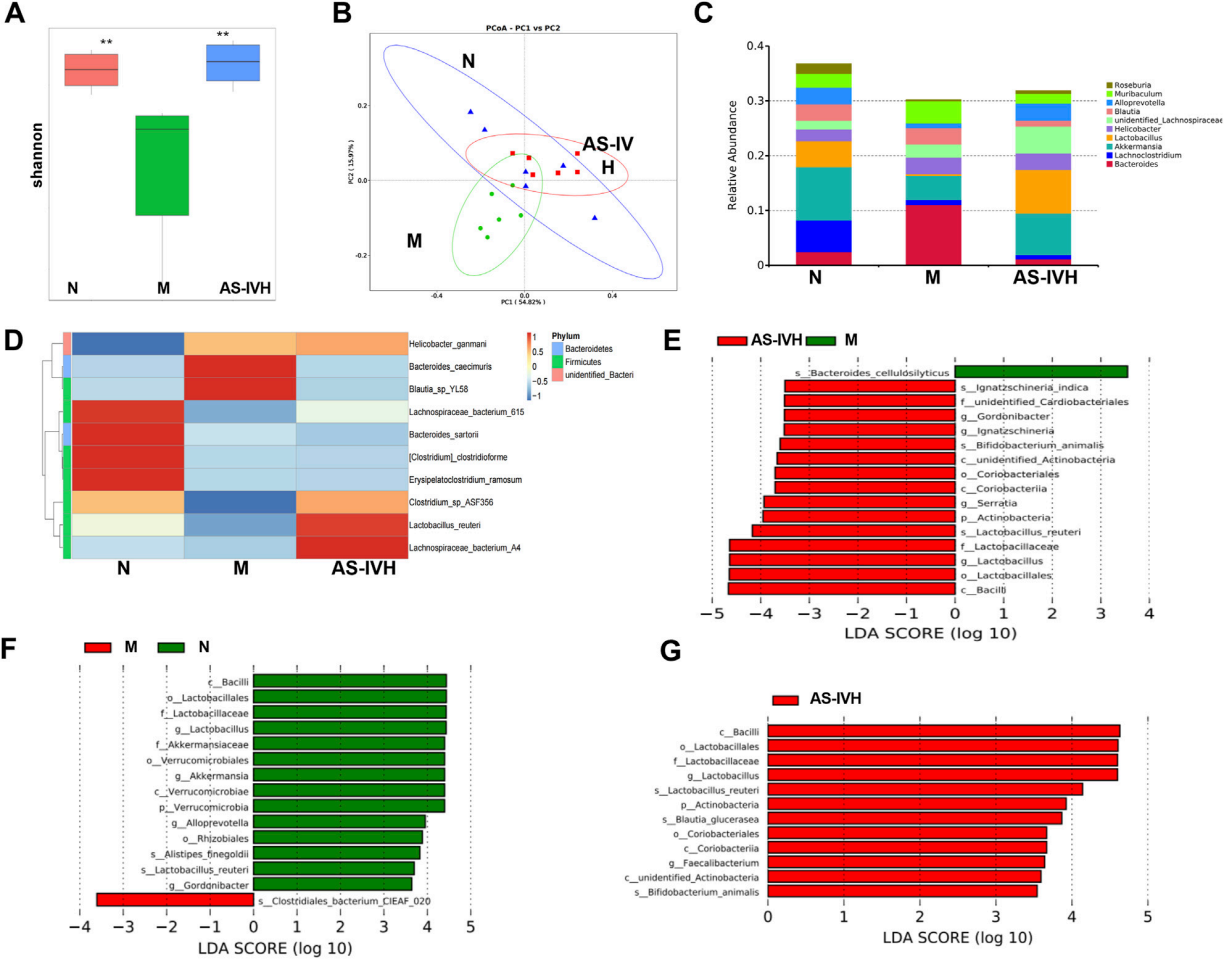
Regulatory effect of AS-IV on the intestinal microbiota. **(A)**. Box plot of the Shannon index, **P < 0.01 compared with the M group. **(B)**. Principal coordinate analysis (PCoA). **(C)**. Bar chart of the top 10 genera. **(D)**. Chart of the clustering analysis of the top 10 species. **(E–G)** Identification of dominant species between groups through LEfSe. The missing group represents the absence of dominant bacteria (E. Group N vs. Group M vs. group AS-IVH; F. Group N vs. Group M; G. Group M vs. group AS-IVH).

Further analysis of the gut microbiota was conducted in the N, M, and AS-IVH groups using 16S rDNA sequencing. AS-IV increased the Shannon index, which was decreased in the M group, and exhibited a colony aggregation pattern similar to that of the N group (Figures 3A, B). The top 10 genera were *Bacteroides, Lachnoclostridium, Akkermansia, Lactobacillus, Helicobacter, unidentified_Lachnospiraceae, Blautia, Alloprevotella, Muribaculum* and *Roseburia* (Figure 3C). Furthermore, the top 10 species were *Lactobacillus reuteri, Bacteroides sartorii, Bacteroides caecimuris, Clostridium clostridioforme, Blautia_sp_YL58, Erysipelatoclostridium ramosum, Lachnospiraceae_bacterium_615, Lachnospiraceae_bacterium_A4, Helicobacter ganmani and Clostridium_sp_ASF356 (* Figure 3D *).* LEfSe revealed that *L. reuteri and Alistipes finegoldii dominated Group N. Group M was dominated by Bacteroides cellulosilyticus and Clostridiales bacterium CIEAF_020. Within the AS-IVH group, a number of dominant species were found, including L. reuteri, Blautia glucerasea, Bifidobacterium animalis and Ignatzschineria indica* (Figures 3E–G).

### 3.4 AS-IV regulated urine metabolism

**FIGURE 4:**
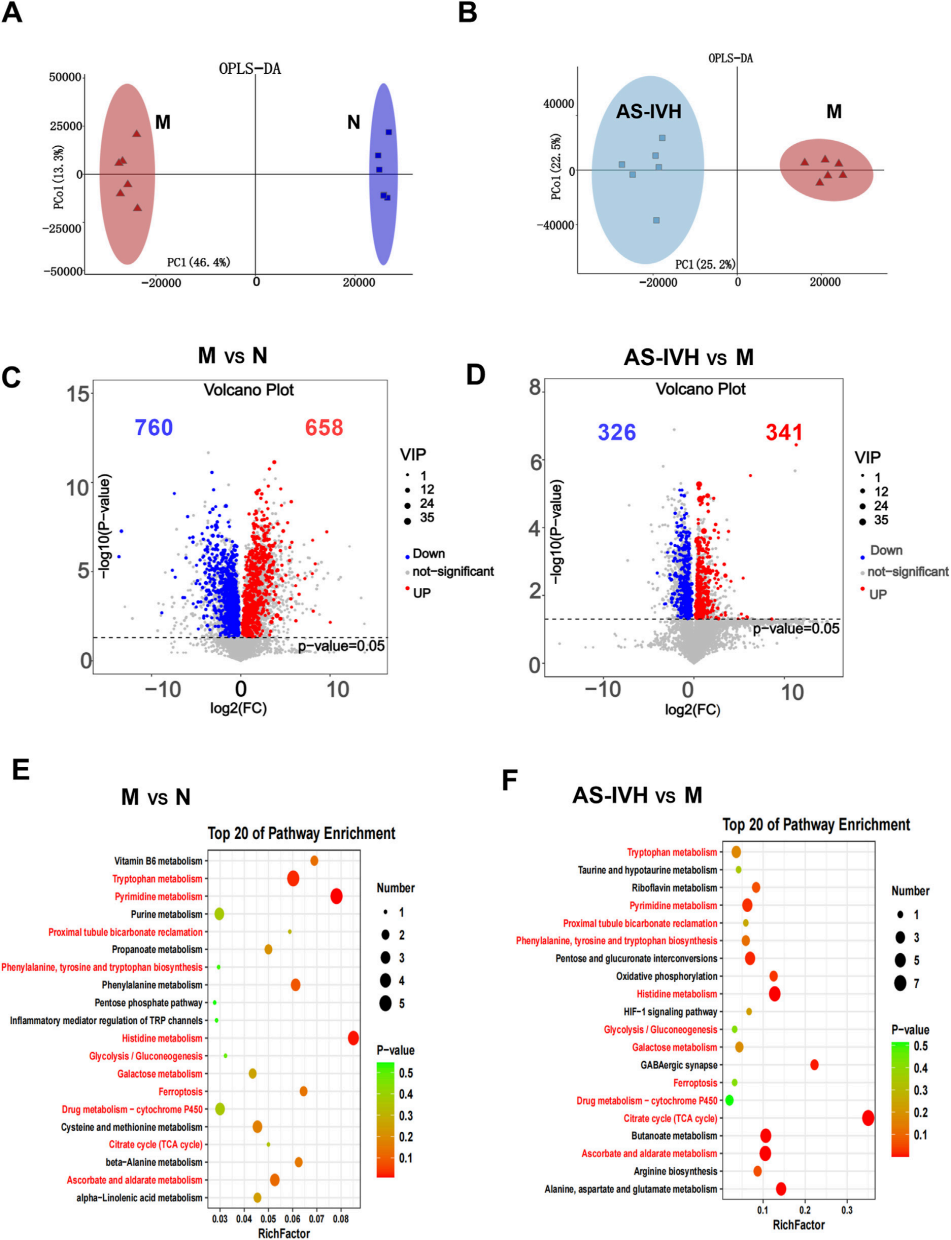
Regulation of urinary metabolism by AS-IV. **(A, B)** Analyses of differentially abundant metabolites via OPLS-DA. **(C, D)** Volcano plots of differentially abundant metabolites. **(E, F)** Top 20 enriched KEGG pathways of the differentially abundant metabolites. The pathway highlighted in red refers to the same pathway in the two enrichment analyses. In all the above comparisons, Group N and Group M were compared, as were Group AS-IVH and Group M.

**FIGURE 5:**
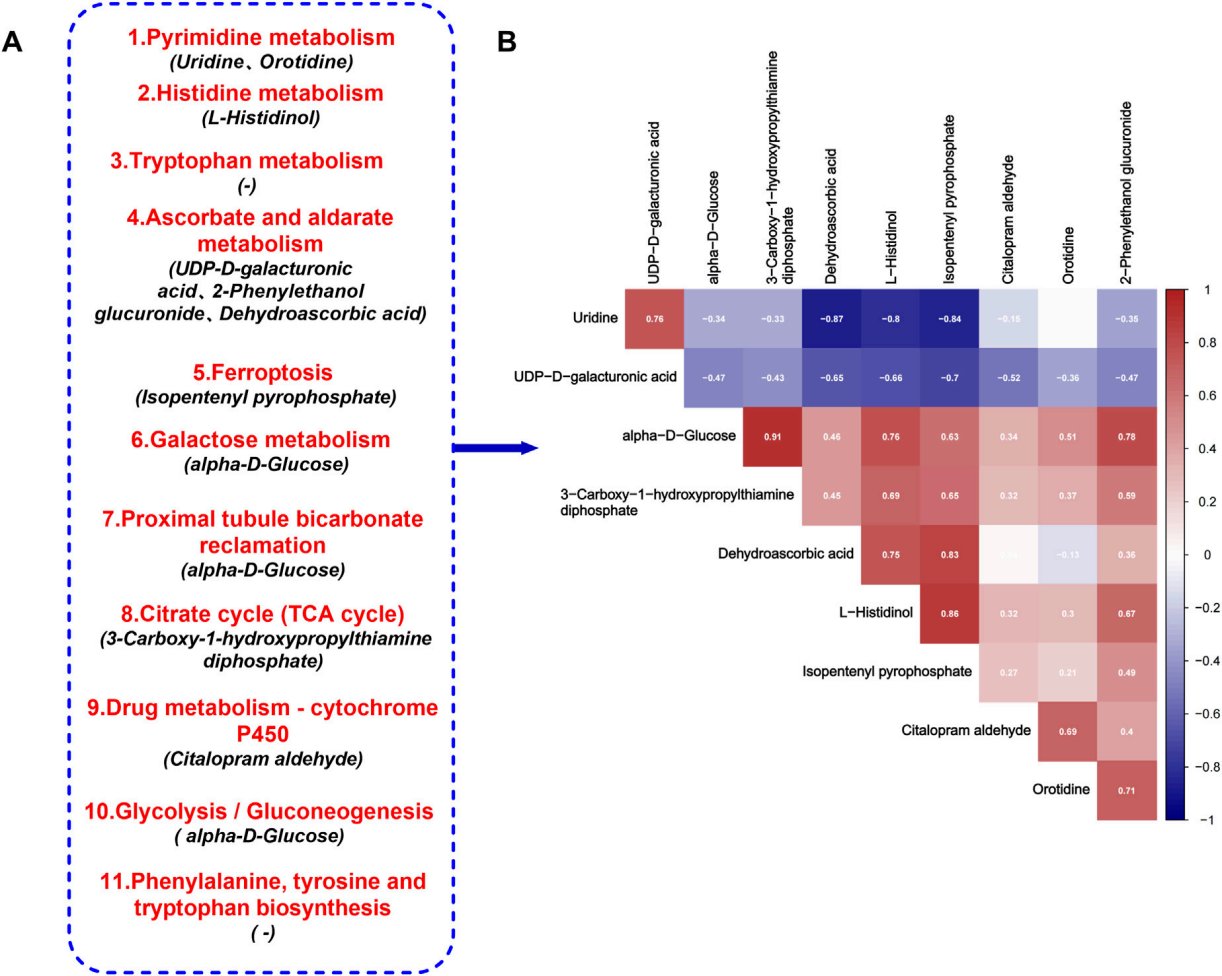
Target metabolic pathways of AS-IV. **(A)** Eleven identical KEGG pathways, among which the differentially abundant metabolites were shared in the two enrichment analyses. **(B)** Correlations of 10 differentially abundant metabolites.

A metabolomic analysis of urine samples from the AS-IVH, N and M groups was conducted. OPLS-DA revealed that the metabolic patterns of the AS-IVH, N and M groups were different, indicating that AS-IV regulated urine metabolism (Figures 4A, B). Compared with Group N, Group M had 1,418 differentially abundant metabolites, including 658 that were upregulated and 760 that were downregulated. Compared with the M group, the AS-IVH group had 667 differentially abundant metabolites, of which 341 were upregulated and 326 were downregulated (Figures 4C, D). A KEGG analysis was subsequently conducted on the differentially abundant metabolites (Figures 4E, F). Eleven pathways were coenriched among the top 20 pathways identified in both enrichment analyses. Among the 11 identical pathways, a total of 10 identical differentially abundant metabolites were observed (Figure 5A). Moreover, metabolites involved in *ascorbate and aldarate metabolism, proximal tubule bicarbonate reclamation, glycolysis/gluconeogenesis, pyrimidine metabolism, the citrate cycle (TCA cycle), histidine metabolism* and *ferroptosis pathways* were highly correlated (Figure 5B).

### 3.5 LncRNAs and mRNAs coexpressed in the kidney after treatment with AS-IV

**FIGURE 6:**
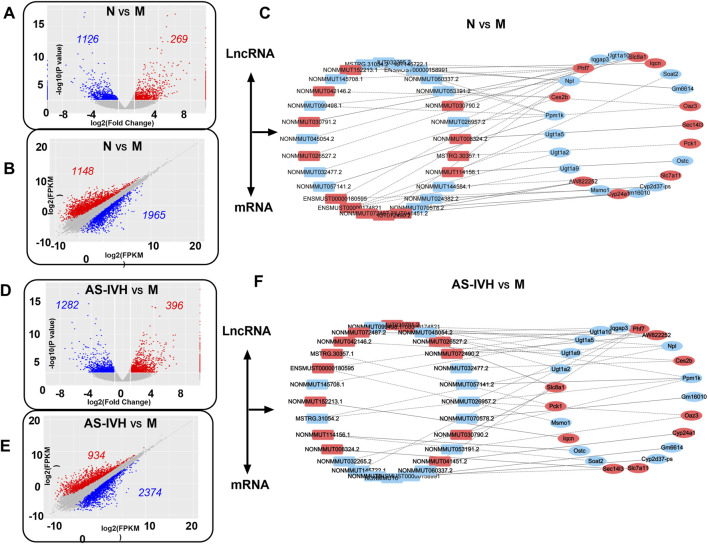
Coexpression analysis of lncRNAs and mRNAs. **(A, D)** Volcano plots of differentially expressed lncRNAs. **(B, E)** Scatter plots of differentially expressed mRNAs. **(C, F)** Coexpression network of lncRNAs and mRNAs. The lncRNAs and mRNAs whose expression was upregulated are presented in red, whereas those whose expression was downregulated are presented in blue. Comparative analyses were conducted between Groups N and M, as well as between groups AS-IVH and M.

**FIGURE 7:**
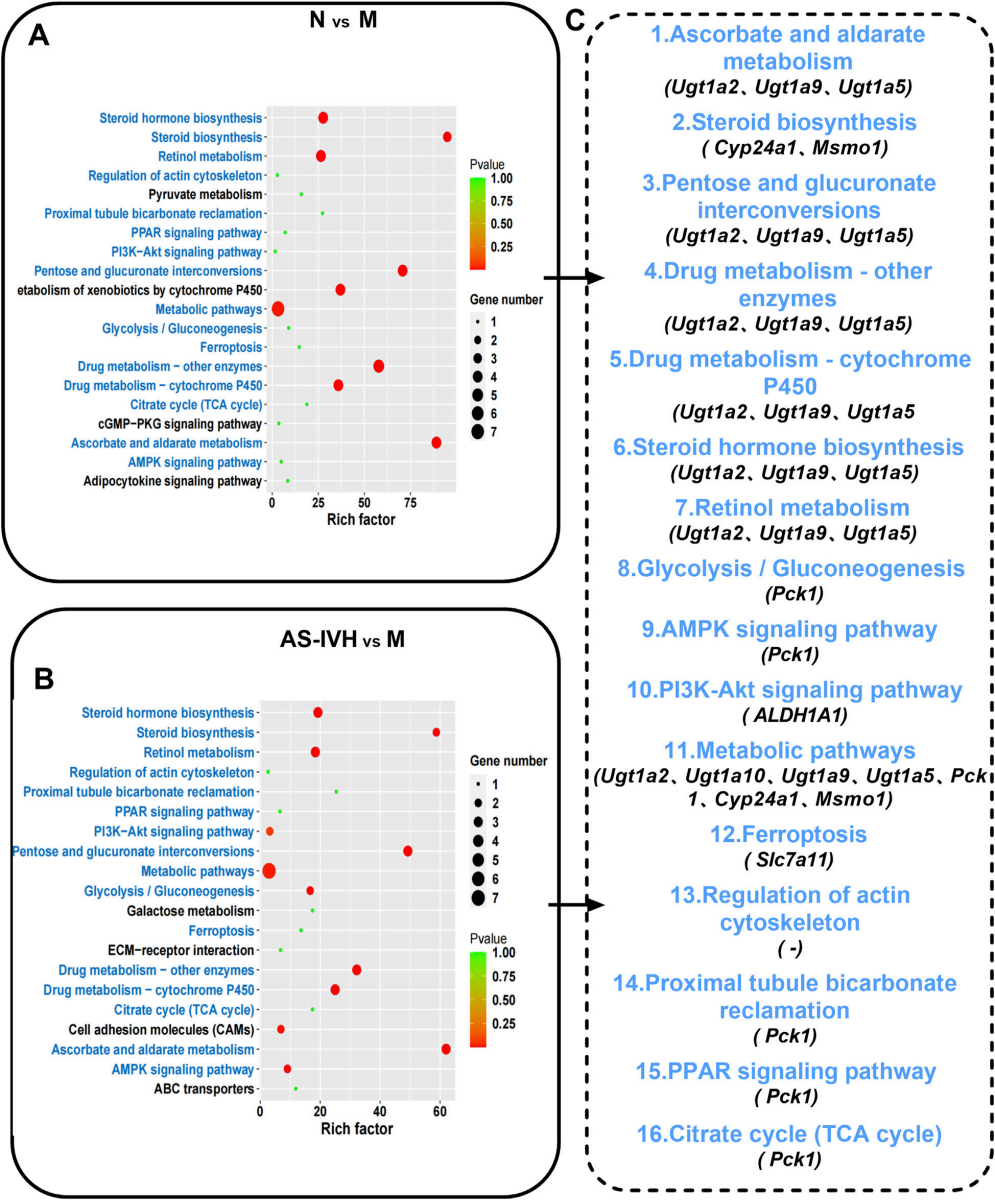
KEGG enrichment analysis of coexpressed mRNAs. **(A, B)** Top 20 enriched KEGG pathways of coexpressed mRNAs (N vs. M; AS-IVH vs. M; blue represents the same pathway). **(C)** Sixteen identical pathways. The mRNAs are shared in the two enrichment analyses.

The kidneys from the AS-IVH, N and M groups were also sequenced to detect lncRNAs and mRNAs. A total of 1,395 lncRNAs were differentially expressed between Groups M and N, with 269 upregulated and 1,126 downregulated lncRNAs. Compared with the M group, the AS-IVH group presented 1,678 differentially expressed lncRNAs, of which 396 were upregulated and 1,282 were downregulated (Figures 6A, D). Among the 3,113 differentially expressed mRNAs in Group N, 1,148 were upregulated, whereas 1965 were downregulated compared with those in Group M. Compared with the M group, the AS-IVH group had 3,308 differentially expressed mRNAs, including 934 upregulated and 2,374 downregulated mRNAs (Figures 6B, E). We further constructed a coexpression network of lncRNAs and mRNAs and performed a KEGG enrichment analysis of the mRNAs within it (Figures 6C, F). Among the top 20 metabolic pathways obtained from the two enrichment analyses, a total of 16 identical pathways were identified, with 8 identical differentially expressed mRNAs predicted by 5 identical or different lncRNAs (Figures 7A–C).

### 3.6 AS-IV regulates the coexpression of the microbiota, transcriptome, and metabolome

**FIGURE 8:**
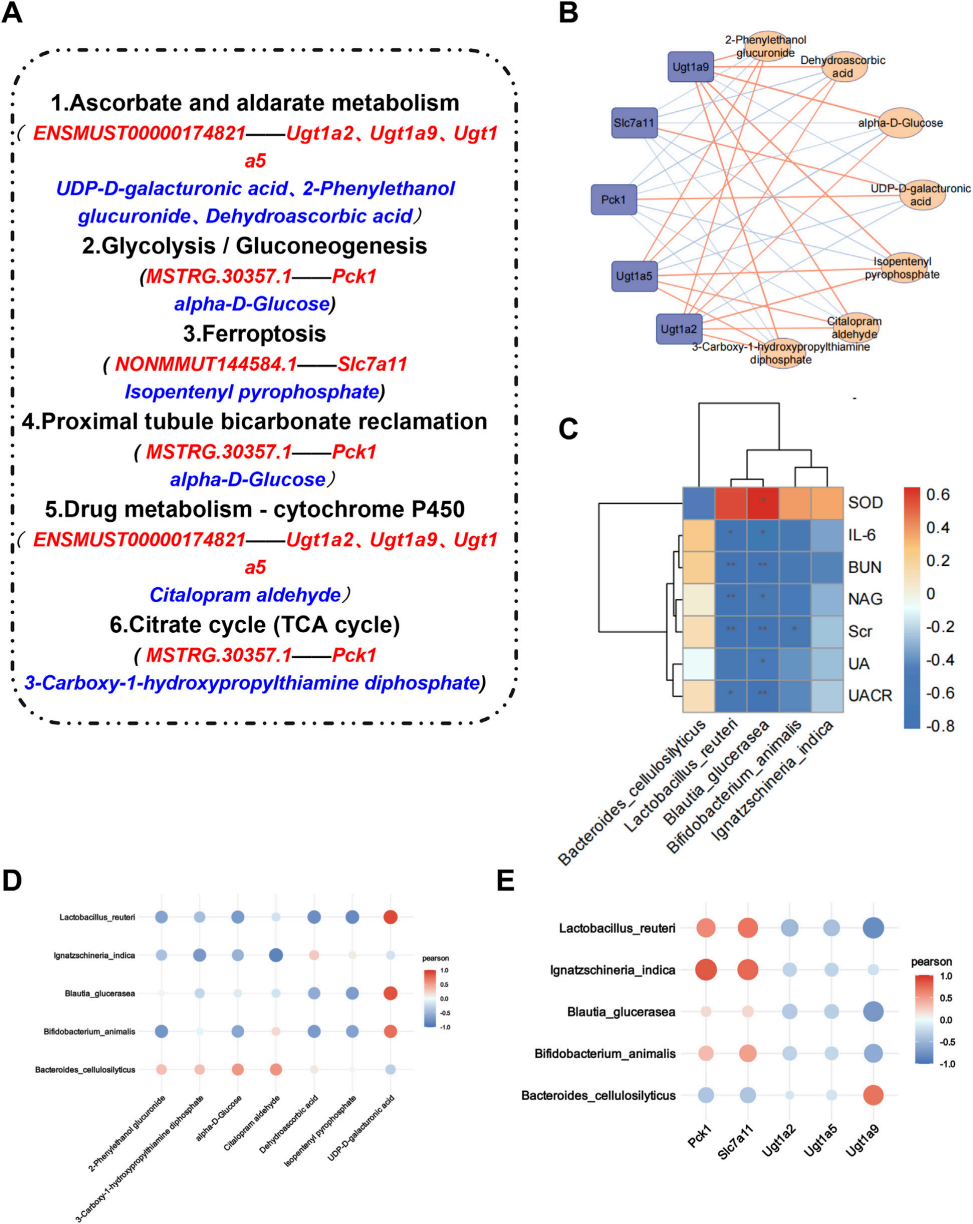
Association analysis of AS-IV target bacteria with target metabolites, target genes, and renal function indicators. **(A)** The 6 pathways involved in the renal transcriptome and urinary metabolome are the same, with red representing the target lncRNAs and mRNAs regulated by it and blue representing the target metabolites in the pathway; **(B)** Gene‒metabolite correlation analysis. Positive correlations are represented by yellow lines, and negative correlations are represented by blue lines. The thicker the line is, the stronger the correlation. **(C)** Correlation analysis between the microbiota and renal function indicators. *p < 0.05 and **p < 0.01. **(D, E)** Correlation analysis of the microbiota with metabolites and genes. The larger the point is, the stronger the correlation.

A correlation analysis was subsequently conducted on the target bacteria, metabolites, and genes regulated by AS-IV. By comparing the renal transcriptome with the urine metabolome, we found that six pathways, including *ascorbate and aldarate metabolism*, *the citrate cycle*, *proximal tubule bicarbonate reclamation*, *glycolysis/gluconeogenesis, drug metabolism–cytochrome P450* and *ferroptosis*, were coenriched. A total of 7 differentially abundant metabolites (Table 1) and 5 differentially expressed genes were identified in the 6 identical pathways. Five genes were targeted and regulated by 3 identical or different lncRNAs (Figure 8A). The correlation analysis revealed strong correlations between metabolites and genes involved in the *citrate cycle*, *ascorbate and aldarate metabolism*, and *glycolysis/gluconeogenesis* pathways (Figure 8B). Furthermore, *L. reuteri* was significantly negatively correlated with the renal function indicators NAG, UACR, IL-6, Scr, and BUN levels; significantly negatively correlated with Ugt1a9 and isopentenyl pyrophosphate levels; and positively correlated with UDP-D-galacturonic acid levels. *Blautia glucerasea* was significantly negatively correlated with NAG, UA, UACR, IL-6, Scr, SOD, and BUN levels and significantly positively correlated with UDP-D-galacturonic acid levels. *Ignatzschineria indica* correlated positively with Pck1 and Slc7a11 levels and negatively with citalopram aldehyde levels (Figures 8C–E).

**TABLE 1:** Seven target metabolites of AS-IV.

Metabolite	Formula	m/z	Retention time (min)	Trend for the change (N:M/AS-IVH:M)	Pathway
3-Carboxy-1-hydroxypropylthiamine diphosphate	C16H25N4O10P2S	508.0585113	4.069316667	↓/↓	Citrate cycle (TCA cycle)
UDP-D-galacturonic acid	C15H22N2O18P2	579.0271684	9.907616667	↑/↑	Ascorbate and aldarate metabolism
2-Phenylethanol glucuronide	C14H18O7	297.0977336	5.145333333	↓/↓	Ascorbate and aldarate metabolism
Dehydroascorbic acid	C6H6O6	173.0088084	0.846833333	↓/↓	Ascorbate and aldarate metabolism
Alpha-D-glucose	C6H12O6	179.0558559	0.757116667	↓/↓	Proximal tubule bicarbonate reclamation; glycolysis/gluconeogenesis
Isopentenyl pyrophosphate	C5H12O7P2	244.9984195	1.31145	↓/↓	Ferroptosis
Citalopram aldehyde	C18H14FNO2	589.196196	4.562283333	↓/↓	Drug metabolism–cytochrome P450

### 3.7 AS-IV inhibited nephrotoxicity

**FIGURE 9:**
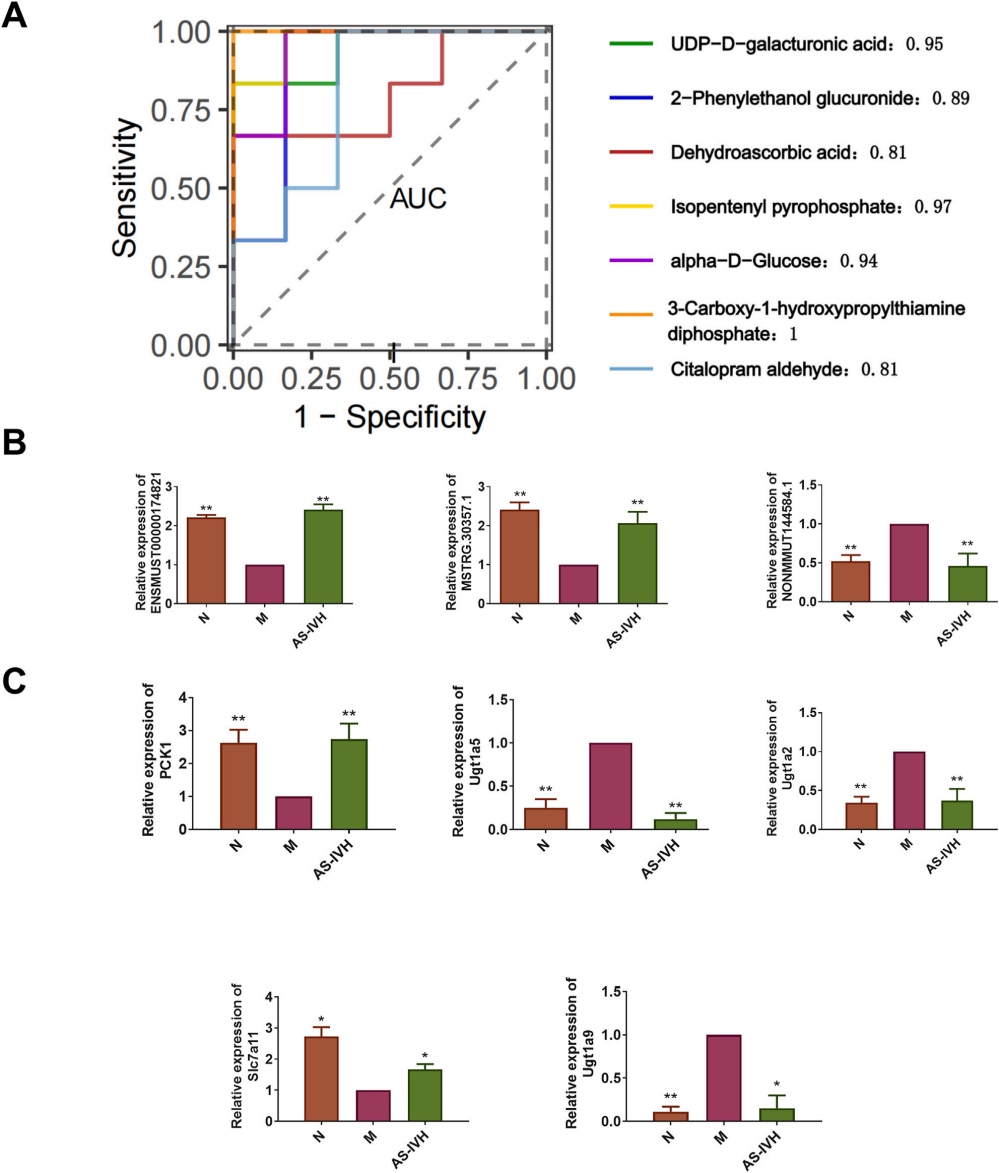
Validation of target genes and metabolites of AS-IV. **(A)** An analysis of the ROC curves for seven target metabolites of AS-IV was performed. **(B)** RT‒qPCR analysis of ENSMUST00000174821, NONMMUT144584.1, and MSTRG.30357.1 expression (n = 3). **(C)** RT‒qPCR analysis of Pck1, Ugt1a2, Ugt1a5, Ugt1a9 and Slc7a11 expression (n = 3). Compared with the M group, *p < 0.05 and **p < 0.01.

**FIGURE 10:**
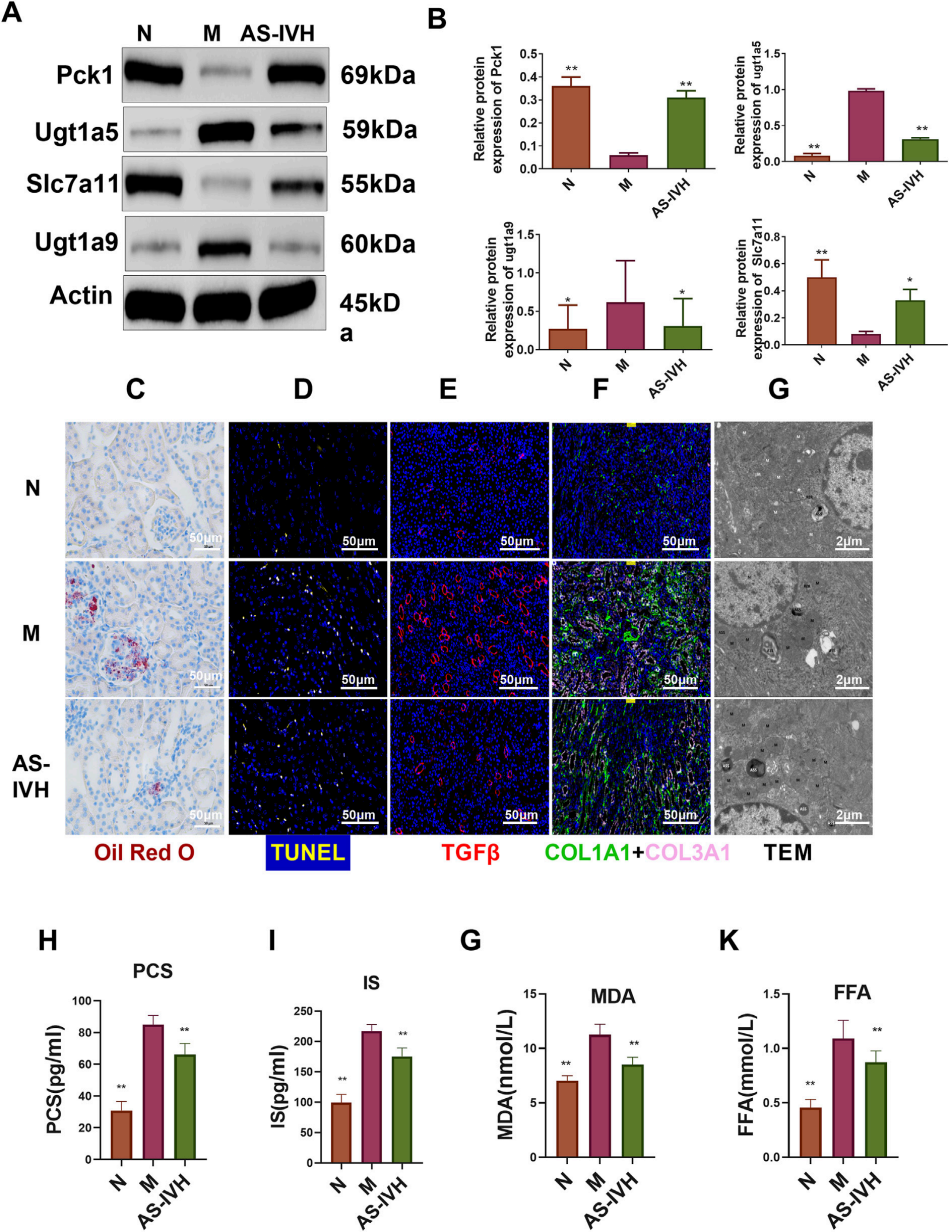
Regulatory effects of AS-IV on nephrotoxicity. **(A)** Western blot bands showing the levels of Pck1, Ugt1a5, Ugt1a9 and Slc7a11. **(B)** Semiquantitative analysis of the proteins. **(C)** Staining of the kidney with Oil Red O, in which red represents lipids (bar = 50 μm, 400×). **(D)** Cells undergoing apoptosis in the kidney, where yellow indicates apoptosis (bar = 50 μm, 400×). **(E)** Immunofluorescence staining for TGFβ (bar = 50 μm, 400×, red represents TGFβ). **(F)** Immunofluorescence staining for COL1A1 and COL3A1 (bar = 50 μm, 400×, pink represents COL1A1 and green represents COL3A1). **(G)** Transmission electron microscopy (TEM) images (8,000×). **(H–K)** Serum p-cresol sulfate (PCS), indoxyl sulfate (IS), malondialdehyde (MDA) and free fatty acid (FFA) contents (n = 8). Compared with the M group, *p < 0.05 and **p < 0.01.

We further validated the target genes and metabolites of AS-IV. All 7 differentially expressed metabolites had high AUC predictability (Figure 9A). The levels of ENSMUST00000174821, Pck1, and Slc7a11 were increased by AS-IV, whereas the levels of NONMMUT144584.1, MSTRG. 30357.1, Ugt1a2, Ugt1a5, and Ugt1a9 were decreased by AS-IV (*P* < 0.01 or *P* < 0.05) (Figures 9B, C). Ugt1a5 and Ugt1a9 protein expression levels were decreased by AS-IV, and Pck1 and Slc7a11 protein expression levels were increased (*P* < 0.01 or *P* < 0.05) (Figures 10A, B). AS-IV improved apoptosis and lipid accumulation and reduced the deposition of TGFβ, COL1A1 and COL3A1 in the renal tissue (Figures 10C–F). Moreover, the number and uneven distribution of mitochondria in the M group decreased, and the arrangement of cristae became disordered and reduced, which improved after the AS-IV intervention (Figure 10G). In addition, AS-IV reduced the levels of the enterotoxins PCS and IS, reduced the levels of MDA and FFA, and alleviated oxidative stress and lipid toxicity (*P* < 0.01) (Figures 10H–K).

## 4 Discussion

Currently, the renal toxicity of CsA remains a major limitation to its clinical application. Fibrosis, which is the primary characteristic of CsA-induced renal toxicity, is difficult to reverse, making early prevention and treatment particularly crucial. Traditional Chinese medicine has shown unique efficacy in the clinical treatment of drug-induced renal injury and improvement of CKD. Research on effective monomers from herbs to advance drug development and application is highly important. As an active constituent of *A. membranaceus*, AS-IV can ameliorate renal injury through multiple mechanisms. With the introduction of the gut‒kidney axis concept, its role in CKD has gradually garnered attention. Alterations in the gut microbiota can exacerbate renal injury by influencing energy metabolism, immune inflammation, oxidative stress, and urinary toxins (He et al., 2024). Studies have shown that AS-IV can improve DKD by modulating the gut microbiota (Lyu et al., 2024). However, the ameliorative effects of AS-IV on CICN and its mechanism of action under the influence of the “gut‒kidney axis” still need to be elucidated.

This study combined an analysis of the gut microbiota, renal transcriptome, and urine metabolome to show that AS-IV improves CICN by regulating gut–transcriptome–metabolome coexpression and revealed a possible specific regulatory mechanism. Changes in the structure and function of the kidneys and colon were observed. In the M group, urine output decreased, proteinuria increased, renal function was damaged, oxidative stress increased, and inflammation worsened. These changes were accompanied by oedema of renal tubular epithelial cells, increased deposition of collagen, and increased intestinal permeability. AS-IV improved the structural and functional damage to varying degrees.

Subsequently, we screened the target bacteria, metabolites, and genes of AS-IV. *Lactobacillus reuteri, Blautia acidophilus, B. animalis* and *I. indica* were identified as the dominant bacteria regulated by AS-IV, especially *L. reuteri*, which ranked 10th in abundance and was the main species that plays a role. By rebuilding the gut microbiota, increasing butyrate production, and inhibiting kidney inflammation, *L. reuteri* has been proven to alleviate cisplatin-induced kidney damage (Hsiao et al., 2021). It can also regulate energy metabolism-related pathways to protect kidney function and prevent acute kidney injury (Yang et al., 2024). *Blautia glucerasea* belongs to the *Blautia* genus and promotes an increase in the levels of short-chain fatty acids, maintains the integrity of the intestinal barrier, exhibits anti-inflammatory properties, and regulates immune responses (Holmberg et al., 2024). *Bifidobacterium animalis* possesses antioxidant activity that regulates oxidative stress and protects cells from oxidative damage (Lin et al., 2022). Supplementation with *B. animalis* can reduce the abundance of harmful bacterial species, decrease toxin levels in rats, and delay the progression of CKD (Wang et al., 2020). Currently, limited reports are available on *I. indica*, with the literature primarily associating it with infections.

The target pathways of the renal transcriptome and urine metabolome were further compared, and a total of 6 pathways were coenriched, including the *citrate cycle, ascorbate and aldarate metabolism, proximal tubule bicarbonate reclamation, glycolysis/gluconeogenesis, drug metabolism–cytochrome P450* and *ferroptosis*. Among them, 7 differentially abundant metabolites, including UDP-D-galacturonic acid, 2-phenylethanol glucuronide, dehydroascorbic acid, isopentenyl pyrophosphate, alpha-D-glucose, 3-carboxy-1-hydroxypropylthiamine diphosphate and citalopram aldehyde, as well as 5 differentially expressed genes, including Ugt1a2, Ugt1a9, Ugt1a5, Pck1 and Slc7a11, were predicted by NONMMUT144584.1, MSTRG.30357.1 and ENSMUST00000174821. A further correlation analysis revealed that *L. reuteri* was significantly correlated with renal function indicators, *ferroptosis* and *ascorbate and aldarate metabolism*. *Lactobacillus reuteri* may be an important initiating factor for AS-IV to improve CICN by regulating the gut‒kidney axis.

After the AS-IV intervention, the content of 3-carboxy-1-hydroxypropylthiamine diphosphate in the *citrate cycle* pathway decreased, and the expression of MSTRG.30357.1 and its target gene Pck1 increased. The main function of the TCA cycle is to generate electrons and provide energy for the electron transport system. In kidney disease, the levels of metabolites in the tricarboxylic acid cycle and enzymes involved in its synthesis are altered, affecting kidney function by regulating immunity, epigenetics, redox reactions, and other processes (Jiménez-Uribe et al., 2021). Tricarboxylic acid cycle metabolites may be used as biomarkers of kidney disease. 3-Carboxy-1-hydroxypropylthiophene is an intermediate in the citric acid cycle and is the second to last step in the synthesis of succinyl CoA. Mitochondrial energy metabolism disorders caused by succinyl CoA deficiency are closely related to heart failure (Takada et al., 2022). Research has shown that a decrease in serum 3-carboxy-1-hydroxypropylthiam levels is closely related to heart failure (Wen et al., 2020). In addition to being a target of MSTRG.30357.1, Pck1 plays a key role in the tricarboxylic acid cycle, which is responsible for providing energy to renal tubular epithelial cells by accelerating cellular respiration and preventing fuel oxidation failure (Verissimo et al., 2023). A proteomic analysis indicated that Pck1 is a biomarker for renal ageing (Yi et al., 2020). The expression of Pck1 is significantly reduced in mice with folate-induced renal interstitial fibrosis and is closely related to renal failure and ECM deposition (Shen et al., 2018). Therefore, AS-IV may regulate the content of 3-carboxy-1-hydroxypropylthiamine diphosphate and the expression of MSTRG.30357.1 and its target gene Pck1 by regulating the citrate cycle, thereby ameliorating CICN.

AS-IV reduced the levels of 2-phenylethanol glucuronide and dehydroascorbic acid involved in *ascorbate and aldarate metabolism*, whereas the level of UDP-D-galacturonic acid increased. The expression of ENSMUST00000174821 was upregulated, whereas the expression of its target genes Ugt1a2, Ugt1a5, and Ugt1a9 was downregulated. The ascorbate and aldarate metabolic pathway protects cells from oxidative damage and plays a significant role in diabetic nephropathy, obesity-related renal damage, and renal fibrosis (Han et al., 2023; Liang and Song, 2024; Chang et al., 2020). In the liver, UDP glucuronosyltransferase produces 2-phenylethanol glucuronide, a natural metabolite of 2-phenylethanol. Glucosylation is used to eliminate toxic substances, drugs, or other substances that cannot be utilized for energy. An elevated 2-phenylethanol glucuronide level in urine is a potential biomarker of depression (Zhang et al., 2021). Dehydroascorbic acid (DHA) is an oxidized form of ascorbic acid that is actively transported into cells via glucose transporters in the endoplasmic reticulum. DHA can accumulate in cells through the autocrine cycle, consuming GSH and ATP, inhibiting glycolysis, and ultimately inducing cell death (Ferrada et al., 2019). High-dose DHA treatment can induce ROS production and promote ferroptosis (Ferrada et al., 2023). UDP-D-galacturonic acid is a type of pyrimidine nucleotide sugar (Ding et al., 2018). UDP glucuronosyltransferases (UGTs) are essential drug-metabolizing enzymes for the metabolism of endogenous substrates and exogenous substances. The expression of the Ugt1a2 gene was reduced in the kidneys of diabetic nephropathy mice (Rubin et al., 2016). Elevated expression of Ugt1a5 is associated with liver and kidney toxicity caused by antibiotic residues (Zhao et al., 2022). In the metabolic transformation and transport process of kidney transplantation, especially in the context of cyclosporine metabolism, genetic variations in the Ugt1a9 gene may lead to kidney damage (Hryniewiecka et al., 2020). AS-IV may ameliorate CICN by regulating the expression of the aforementioned genes and metabolites through the regulation of ascorbate and alderate metabolism.

After the intervention with AS-IV, the content of alpha-D-glucose decreased, which belongs to the pathway of glycolysis/gluconeogenesis and proximal tubule bicarbonate reclamation, and Pck1 is also a target gene for both pathways. In proximal tubular cells, CKD progression is associated with the conversion of fatty acid oxidation to glycolysis. The stage-dependent loss of renal tubular gluconeogenesis is a key feature of CKD and may lead to systemic and local metabolic complications (Dalga et al., 2023; Zhao et al., 2023). In addition, proximal tubule bicarbonate reclamation is involved in contrast material-induced acute kidney injury and lupus nephritis (Deng et al., 2023; Ghasemi et al., 2021). Alpha-D-glucose is the alpha isomer of D-glucose, a fundamental metabolite found in all living organisms, from bacteria to plants to humans, and is involved in energy metabolism (Cifuente et al., 2024). AS-IV may regulate the expression of MSTRG.30357.1 and its target gene Pck1, affecting alpha-D-glucose metabolism levels thereby regulating glycolysis/gluconeogenesis and proximal tube bicarbonate metabolism, promoting renal energy metabolism, and reducing renal tissue apoptosis.

After the AS-IV intervention, the content of isopentenyl pyrophosphate in the *ferroptosis* pathway decreased, the expression of NONMMUT144584.1 decreased, and the expression of its target gene Slc7a11 increased. Isopentenyl pyrophosphate has previously been reported to act as a novel antiviral agent that inhibits the TRPV3 and TRPA1 ion channels (Bang et al., 2011). In different kidney injury models, inhibiting the expression of Slc7a11 promotes ferroptosis, exacerbates lipid peroxidation, and accelerates renal fibrosis (Yang and Guo, 2023; Ji et al., 2023). Furthermore, a decrease in serum Slc7a11 levels is independently associated with vascular calcification in maintenance peritoneal dialysis patients (Wu et al., 2024). In addition, after the AS-IV intervention, the expression of citalopram aldehyde in the *drug metabolism–cytochrome P450* pathway decreased, and ENSMUST00000174821 and its target genes Ugt1a2, Ugt1a5, and Ugt1a9 also belong to this pathway. Citalopram aldehyde is a metabolite of citalopram, which can protect nerves by attenuating oxidative stress, inflammation, and cell apoptosis (Gupta et al., 2018). AS-IV may improve the excretion of isopentenyl pyrophosphate and citalopram aldehyde, as well as the expression of NONMMUT144584.1, ENSMUST00000174821, Ugt1a2, Ugt1a5, Ugt1a9, and Slc7a11, by regulating the *ferroptosis* and *drug metabolism–cytochrome P450* pathways, thus protecting renal function.

The AUC predictions for all seven differentially expressed metabolites were very high, and all five mRNAs and three lncRNAs were highly consistent with those obtained by sequencing. After the AS-IV intervention, renal tissue apoptosis and lipid accumulation decreased; the deposition of TGFβ, COL1A1 and COL3A1 decreased; and the damage to mitochondria was alleviated. In addition, AS-IV reduced the levels of the enterotoxins PCS and IS, reduced the levels of MDA and FFA, and alleviated oxidative stress and lipid toxicity. This regulation may be closely related to the regulation of the target mRNAs and metabolites described above in the energy metabolism pathway driven by dominant bacteria such as *L. reuteri*, *B. glucerasea*, *B. animalis*, and *I. indica*, especially *L. reuteri*.

**FIGURE 11:**
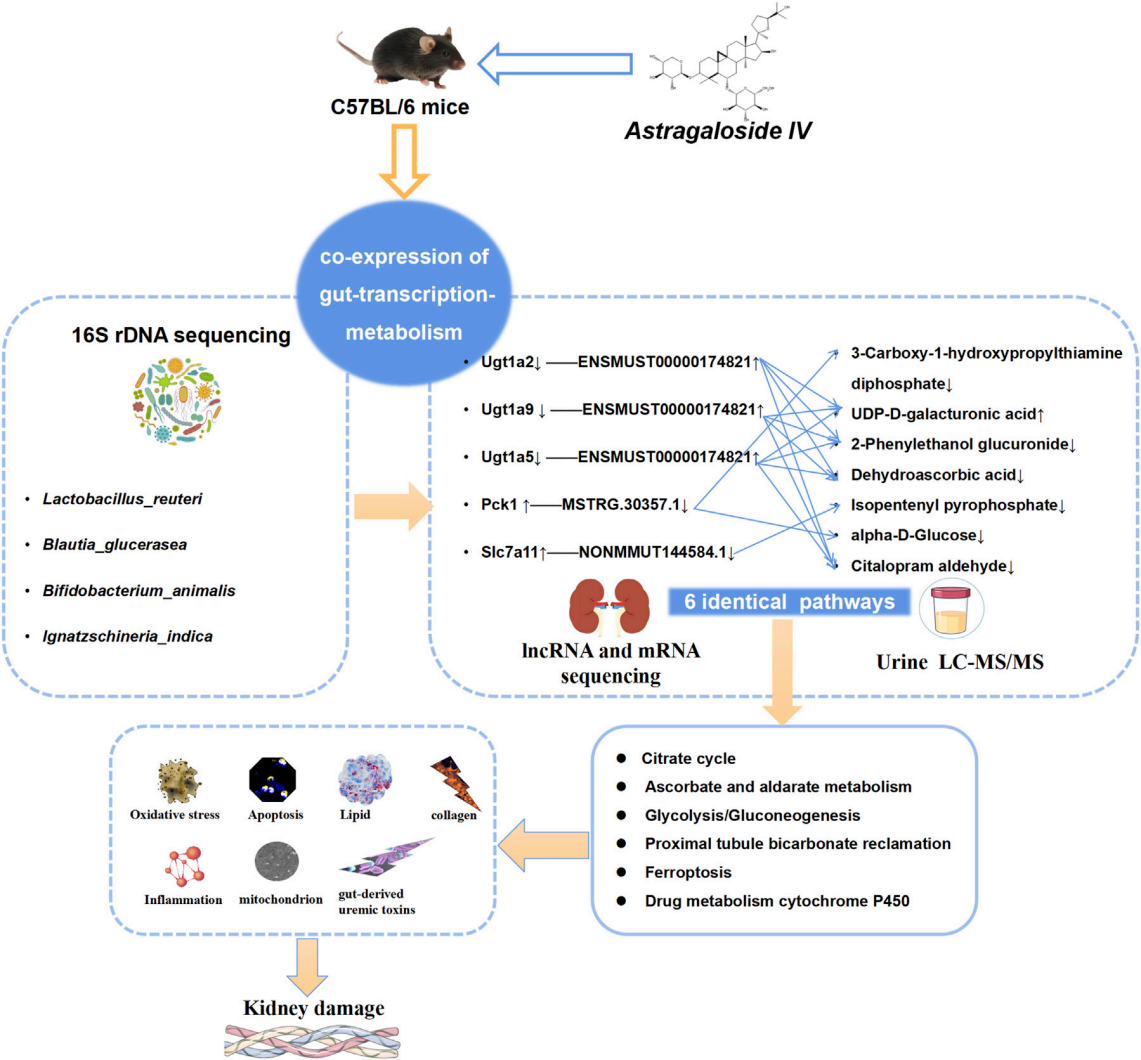
AS-IV regulates “gut microbiota–genes–metabolites” to ameliorate CsA nephrotoxicity. In the “gut microbiota–gene‒metabolite” coexpression network, the gut microbiota regulates target genes and thus target metabolites. *Lactobacillus reuteri* is the main driver. The regulation of target mRNAs and target metabolites in six pathways related to energy metabolism, including the citrate cycle, ascorbate and alderate metabolism, proximal tube bicarbonate metabolism, glycolysis/gluconeogenesis, ferroptosis, and drug metabolism–cytochrome P450, is an important mechanism.

Our research results indicate that AS-IV can ameliorate CICN by regulating the gut microbiota to regulate the levels of target genes and target metabolites. *Lactobacillus reuteri* serves as the primary driving force. Six pathways associated with energy metabolism, including the citrate cycle, ascorbate and alderate metabolism, proximal tube bicarbonate metabolism, glycolysis/gluconeogenesis, ferroptosis, and drug metabolism–cytochrome P450, serve as significant feedback mechanisms (Figure 11).

## Data Availability

The original contributions presented in the study are included in the article/Supplementary Material, further inquiries can be directed to the corresponding author.

## Abbreviations

Pck1, phosphoenolpyruvate carboxykinase 1; Ugt1a2, UDP glucuronosyltransferase 1 family, polypeptide A2; Ugt1a5, UDP glucuronosyltransferase 1 family, polypeptide A5; Ugt1a9, UDP glucuronosyltransferase 1 family, polypeptide A9; Slc7a11, solute carrier family 7, member 11; FN, fibronectin; BUN, blood urea nitrogen; Scr, serum creatinine; UA, uric acid; IL-6, interleukin-6; NAG, N-acetyl-beta-D-glucosaminidase; SOD, superoxide dismutase; MDA, malondialdehyde; FFA, free fatty acid; PCS, p-cresol sulfate; IS, indoxyl sulfate.
