# Supplementary material for: The gut–kidney axis is regulated by astragaloside IV to inhibit cyclosporine A-induced nephrotoxicity
Source: Front Pharmacol. 2025 Jan 27;16:1518481. doi: 10.3389/fphar.2025.1518481 (PMC11807982; doi:10.3389/fphar.2025.1518481)
Supplement: Supplementary file 1 [file Table1.docx]

**Table S1. List of primer sequences (5' to 3’) employed for assessing mRNA expression level.**

| Gene | Strand | Primer sequence (5’-3’) |
| --- | --- | --- |
| GAPDH | Forward | CCTCGTCCCGTAGACAAAATG |
|  | Reverse | TGAGGTCAATGAAGGGGTCGT |
| Pck1 | Forward | CAACCCTGAGCTGACCCTGAT |
|  | Reverse | GGTTAGTTATGCCCAGGATCAGC |
| Ugt1a2 | Forward | CATGGAAACTCGGGGAGCTG |
|  | Reverse | ATTCCACCCAGAACACAGCC |
| Ugt1a5 | Forward | AGTTCCGATGGTGATGATGCC |
|  | Reverse | AGTCATTTCAAGGACATTCAGGGT |
| Ugt1a9 | Forward | TGGGATCAACTGCCTCCAGAAG |
|  | Reverse | GCCAGAGTGTGTGATGAATGC |
| Slc7a11 | Forward | ACCTGCAGGGCAATATGAGC |
|  | Reverse | CCCCTCAGCAAAGTGATCTTCT |
| ENSMUST00000174821 | Forward | TGTGTATTGAGGCTTCAGGTGCTA |
|  | Reverse | CTGTTGTGTGTGTTCGGTCCC |
| NONMMUT144584.1 | Forward | GTTTCTCCCAGGCTACGACTCT |
|  | Reverse | GTTGGTGGACATTTGGAAAGTAAG |
| MSTRG.30357.1 | Forward | AAGGTAAGGAAGGGCGGTGTA |
|  | Reverse | ATGCCTGTCTGTCCCATTGTC |
